# Immunoexpression of stem cell markers CD44, CD133, ALDH1-2, and LGR5 in colorectal carcinoma and their association with clinicopathological tumor characteristics

**DOI:** 10.31744/einstein_journal/2026AO2153

**Published:** 2026-05-27

**Authors:** Karine Corcione Turke, Cinthia dos Santos, Carla Pagliari, Giovanna Maria Gimenez Testa, Thérèse Rachell Theodoro, Bianca Bianco, Jaques Waisberg

**Affiliations:** 1 Centro Universitário FMABC Santo André SP Brazil Centro Universitário FMABC, Santo André, SP, Brazil.; 2 Universidade de São Paulo Faculdade de Medicina São Paulo SP Brazil Faculdade de Medicina, Universidade de São Paulo, São Paulo, SP, Brazil.; 3 Hospital do Servidor Público Estadual "Francisco Morato de Oliveira" Department of Surgery, Gastroenterology Surgical Section São Paulo SP Brazil Department of Surgery, Gastroenterology Surgical Section, Hospital do Servidor Público Estadual "Francisco Morato de Oliveira", São Paulo, SP, Brazil.

**Keywords:** Neoplastic stem cells, Biomarkers, tumor, Colorectal neoplasms, Immunohistochemistry, Rectal neoplasms, Colonic neoplasms, Neoplasm metastasis

## Abstract

**Objective:**

To investigate the association of CD44, CD133, ALDH1-2, and LGR5 expression with clinicopathological tumor variables.

**Methods:**

Twenty-eight adult patients with colorectal carcinoma who underwent curative surgery were included in the study. Paraffin-embedded samples were subjected to immunohistochemical staining with anti-CD44, anti-CD133, anti-ADLH1-2, and anti-LGR5 antibodies, and the results were compared with the patients’ clinicopathological variables. P-values <0.05 were considered significant.

**Results:**

Fifteen patients (53.6%) were male. The mean age was 66.8±13.01 (range: 38 to 88) years. The neoplasm was located in the rectum in 17 (60.7%) patients and colon in 11 (39.3%) patients. The mean tumor size was 4.3 (1.0–10.0) cm. The degree of cellular differentiation was moderate in 27 (94.4%) patients. The tumors presented with parietal invasion at the T1 level in two (7.1%) patients, T2 in seven (25%), and T3 in 19 (67.8%). Vascular invasion was present in nine (32.1%) patients and perineural invasion in five (17.8%). Lymph node involvement was observed in 11 patients (39.3%). ALDH1-5 and LGR5 proteins were significantly increased in patients >60 years of age, in the rectum and right colon, and in the presence of lymph node involvement. CD44 protein expression was increased in tumors of the left colon.

**Conclusion:**

Increased tissue levels of ALDH1-2 and LGR5 were related to increased aggressiveness of colorectal carcinoma.

## In Brief

Characteristics attributed to stem cells can explain both unrestricted and differentiated growth patterns detectable in malignant tumors. Turke et al. reported that cancer stem cell markers are associated with greater tumor aggressiveness.

## Highlights

■ Colorectal carcinoma originates from within cancer stem cells of the large intestine.
■ Tissue levels of ALDH1-2 and LGR5 proteins were associated with greater tumor aggressiveness.
■ Discovery of signaling pathways can improve cancer treatments that control cancer stem cells.

## Graphical abstract

**Figure:**
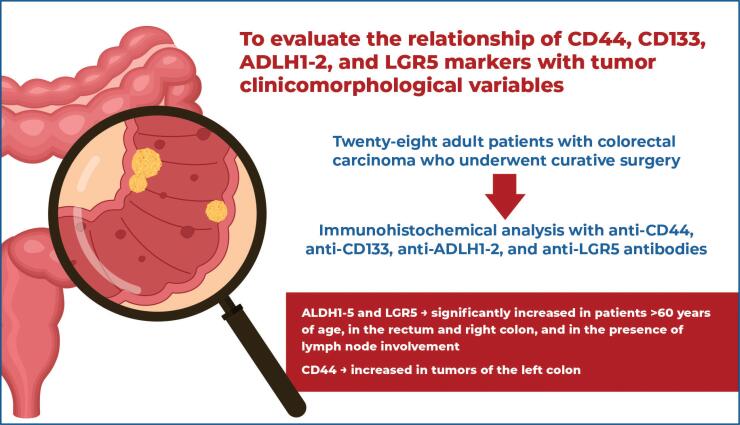

## INTRODUCTION

In colorectal carcinoma (CRC), although improved treatment strategies involving surgery, chemotherapy, and radiotherapy have increased overall survival rates in early stages, 40%–50% of all patients with CRC present with metastasis at the time of diagnosis or as recurrent disease after intended curative therapy.^(1–3)^

However, the survival rate of patients with advanced and metastatic CRC has not changed significantly in recent decades.^(4)^ This scenario is considered sufficient justification for investigating the reasons for CRC resistance to currently available therapeutic procedures. One such line of research is investigation into the roles of cancer stem cells (CSCs).^(5,6)^ The three characteristics attributed to stem cell self-renewal, namely proliferation, and differentiation capacity, can explain the unrestricted and differentiated growth patterns detectable in benign and malignant tumors.^(4)^

The study of CSCs is of medical importance because the homeostatic mechanisms of stem cell proliferation involve the same processes that are dysregulated during carcinogenesis. The discovery of these signaling pathways can improve cancer treatments that rely on the control of CSC proliferation.^(5–7)^ Patients with simultaneous expression of stem cell genes may have a tenfold increased risk of developing colon cancer compared to those with low expression levels.^(8,9)^

Since CSCs have been implicated in the formation of CRC, therapeutic interventions that target only the main tumor mass are likely to have little success or, at best, leave the patient at a high risk of developing carcinoma recurrence.^(10–13)^

CD133 is one of the best-known surface markers of SCs (stem cells) and has been described as an initiator of tumor growth.^(14,15)^ Increased CD133 expression has been shown to be positively correlated with higher T (size and extent in the intestinal wall of the primary tumor) in the TNM classification, as well as lymphatic and vascular invasion, and worse prognosis in colorectal cancer, where it may serve as a therapeutic target.^(16–19)^

CD44, a surface marker present on CSCs, has been found to be associated with increased invasion and metastasis of colorectal cancer. In addition to CD133, its overexpression is associated with increased tumor growth and anti-apoptotic properties. CD133 has prognostic value in colorectal cancer with liver metastasis, where it represents an independent factor associated with survival.^(1, 20-24)^

Aldehyde dehydrogenase isoform 1 (ALDH1) is an intracellular isoform of the aldehyde dehydrogenase enzyme that catalyzes the conversion of aldehyde to carboxylic acid, mediates control over differentiation pathways, and acts in the self-protection of normal stem cells.^(25)^ ALDH1 is commonly used as a marker for the identification of non-tumor stem cells and CSCs in different types of cancers, including breast cancer, pancreatic cancer, prostate cancer, lung cancer, leukemia, multiple myeloma, melanoma, and liver cancer.^(26)^ Increased expression of ALDH1 in colorectal cancer tissue samples has been shown to be associated with poor differentiation (high grade) and metastasis. The detoxifying capacity of ALDH1 may contribute to the longevity of CSCs and protect them against oxidative damage.^(27)^

LGR5, a known target of the Wnt signaling pathway, is a potential marker of stem cells in the small intestine and colon.^(28–30)^ LGR5 is closely associated with tumorigenesis and tumor invasion in CRC and is a relevant CSC marker.^(31–33)^ LGR5 overexpression also correlates with higher levels of lymph node metastasis.^(34,35)^

## OBJECTIVE

To investigate the association of CD44, CD133, ALDH1-2, and LGR5 expression with clinicopathological tumor variables.

## METHODS

The study was conducted according to the ethical standards established by the World Medical Association's Declaration of Helsinki, adopted in 1964 and revised in 2008. This study was registered with the Research Ethics Committee of the (*Centro Universitário FMABC*, under number 021/08), and its conduct followed the principles of good clinical practice.

This investigation employed an observational, longitudinal, and analytical design with retrospective data collection. The study included patients who underwent surgery at *Hospital do Servidor Público Estadual "Francisco Morato de Oliveira"* (São Paulo, Brazil).

The study included 28 adult patients of both sexes and diverse ethnicities, with histologically proven CRC, who had undergone elective curative surgical treatment more than 5 years previously.

Patients with colorectal polyposis syndrome, hereditary non-polyposis CRC, colorectal neoplasia other than carcinoma, or inflammatory bowel disease were excluded. Clinicopathological data were obtained by reviewing patients’ medical records. The following clinicopathological data were analyzed: age; sex; neoplasm location; tumor size; tumor differentiation grade; vascular, perineural, and lymphatic infiltration; and tumor stage according to the TNM classification of the AJCC Cancer Staging Manual (10th edition). Only cases in which pathological examination identified at least 12 lymph nodes without metastatic invasion were considered N0.

Tissue samples from the neoplastic lesions of each patient represented CRC tissues. Carcinoma tissues were obtained from the most representative lesion areas. All of the tissue samples were fixed in 10% buffered formalin and embedded in paraffin.

Tumors were divided into low-grade (well and moderately differentiated) and high-grade (poorly differentiated and undifferentiated) according to the College of American Pathologists standards.

We evaluated the immunohistochemical tissue expression data for the stem cell marker proteins CD44, CD133, CD44, ALDH1-2, and LGR5 in all tissue samples. Immunohistochemical assays were performed at the Multiuser Laboratory of the Department of Morphology and Physiology, *Centro Universitário FMABC* (Santo André, SP, Brazil). Two pathologists independently reviewed the anatomopathological data of all included cases.

Histological sections of 4-*μ*m thickness were obtained and deposited on slides that had been treated with 3-aminopropyl-triethoxysilane adhesive (Sigma, St. Louis, MO, USA). Initially, the slides with histological sections were placed in an oven at 60°C for 24 h to improve tissue adhesion. Deparaffinization was performed in three xylene baths at room temperature for 5 min each, followed by three ethanol baths: absolute (100%), 95%, and 70% (with the slides submerged), and finally, a 100% ethanol bath. Distilled water was added to complete tissue rehydration. The slides were washed in running water for 5 min, subjected to heat (antigen retrieval) in three 1-min microwave baths, and submerged in 10-mM citrate buffer, pH 6.0. After cooling for 20 min at room temperature and subsequent washing under running water for 5 min, endogenous peroxidase was blocked using a 3% hydrogen peroxide solution at 10V in two 20-min baths. The slides were again washed in running water for 5 min and subsequently placed in three 3-min baths with 1X phosphate buffered saline (PBS) buffer, pH 7.2–7.6. For the IHC method, primary monoclonal antibodies against ALDH1, CD44, CD133, and LGR5 (Santa Cruz Biotechnology Inc., Santa Cruz, CA, USA) were used at a dilution of 1:100. All antibodies are recommended for detecting the respective proteins in human tissues. Primary antibodies were diluted in 1X PBS buffer containing 1% bovine serum albumin and incubated in a humidified chamber at 4°C (refrigerator) for 16–18 h (overnight). Immunostaining was performed using the avidin–biotin–peroxidase complex, following the protocol described by the manufacturer for the LSAB+System-HRP kit (Dako North America, Inc., CA, USA), and 3,3’-diaminobenzidine as the liquid chromogen - DAB + Substrate Chromogen System (Dako North America, Inc., CA, USA). A slide containing histological sections that had been proven positive for the studied antibodies was used as the positive control. A similar slide was used as the negative control, and the primary antibody was subtracted from the reaction. The slides were viewed under a Nikon Eclipse® TS100 light optical microscope (Nikon Inc., Tokyo, Japan) to identify the areas that best represented the immunostaining.

In each case, immunostaining was quantified using computer-assisted digital image analysis to determine the intensity of expression (ItE) in *μ*.o./*μ*m². For each case, 640 × 480-pixel photomicrographs were obtained from consecutive and non-coincident fields at 400X magnification under an optical microscope using a Nikon Coolpix 4300® digital camera. The images obtained were analyzed using image processing and the ImageJ® Analysis System (Softium Informática®, São Paulo, Brazil), adjusted to the micrometer scale (*μ*m). Analysis of the immunohistochemical reactions on the slides was semi-quantitative and evaluation of the immunoexpression of the studied proteins was performed according to classification of the results of the immunohistochemical assays, exclusively as positive (protein expression) or negative (absence of protein expression). A result was considered positive when there was unequivocal deposition of a chromogenic product on the cytoplasmic membrane or in the cytoplasm of neoplastic CRC cells in tissue samples subjected to the action of anti-protein monoclonal antibodies. The result was considered negative in samples without any labeling on the membrane or cytoplasm of CRC cells. The immunostaining results were semi-quantitatively based on the ItE analyzed in 12 fields of each of the ten images obtained from each CRC tissue. All slides were analyzed by two well-trained and independent investigators who considered only the intensity of immunostaining (or immunolabeling) using the ImageJ program (NIH ImageJ, US National Institutes of Health, Bethesda, Maryland, USA) without access to or knowledge of the pathological data. In cases of discrepancies in evaluation, the slides were re-evaluated under a dual-eyepiece microscope and both investigators reached a consensus. When staining was not uniform, a comment on heterogeneity was recorded.

Quantitative variables are described as medians and ranges (the difference between the maximum and minimum values). Absolute and relative frequencies were used as qualitative variables. Distributions were defined as nonparametric using the Shapiro–Wilk test. The unpaired t-test with Welch's correction was used for comparisons between two groups, as well as for comparisons between three or more groups. The Kruskal–Wallis test and Dunn's auxiliary test were used for subgroup comparisons. All analyses were performed using Prism software version 5.0 (GraphPad Prism Software Inc., California, USA), adopting a 5% level of statistical significance (p≤0.05).

## RESULTS

Among the 28 patients in this study, 15 (53.6%) were male and 13 (46.4%) were female. The mean age was 66.8±13.01 (range: 38–88) years, and the median age was 69.5 years. All of the patients were Caucasian. The neoplasm was located in the rectum in 17 (60.7%) patients and in the colon in 11 (39.3%) patients. The mean tumor size along its longest axis was 4.3cm (1.0-10.0cm). The degree of cellular differentiation was moderate in 26 (92.9%) cases, and the neoplasm was well-differentiated in only two (7.1%) patients. The tumors presented with parietal invasion at the T1 level in two (7.1%) patients, T2 in seven (25%), and T3 in 19 (67.8%). Vascular invasion was present in nine (32.1%) patients, whereas perineural invasion was observed in five (17.8%) patients. Metastatic lymph node involvement was observed in 11 patients (39.3%) (Table 1).

**Table 1:** Biodemographic and morphological characteristics of patients with colorectal carcinoma

Case	Age	Sex	Site	Size (cm)	Histological differentiation	Wall penetration	Vascular invasion	Perineural invasion	Lymph node metastasis
1	55	M	Rectum	1.7	Moderate	T3	No	No	No
2	65	M	Colon	9.0	Moderate	T3	No	No	No
3	48	F	Rectum	3.5	Moderate	T3	Yes	No	No
4	48	M	Reto	6.0	Moderate	T2	No	No	No
5	60	F	Rectum	4.0	Moderate	T3	No	No	Yes
6	75	M	Rectum	3.5	Moderate	T3	No	Yes	No
7	88	F	Rectum	1.0	Well	T2	No	No	No
8	76	M	Colon	6.5	Moderate	T3	Yes	No	No
9	38	M	Rectum	5.0	Moderate	T3	Yes	Yes	Yes
10	73	M	Rectum	3.0	Moderate	T3	No	No	No
11	67	F	Rectum	9.0	Moderate	T3	No	Yes	Yes
12	76	F	Colon	3.0	Moderate	T3	No	No	Yes
13	77	M	Rectum	3.5	Moderate	T3	No	No	Yes
14	77	M	Rectum	5.5	Moderate	T3	Yes	No	Yes
15	79	M	Colon	3.0	Moderate	T3	Yes	Yes	Yes
16	74	F	Colon	4.0	Moderate	T2	No	No	Yes
17	72	F	Rectum	2.0	Moderate	T2	No	No	No
18	64	F	Rectum	4.0	Moderate	T2	Yes	No	Yes
19	82	M	Rectum	4.0	Moderate	T2	Yes	No	No
20	55	F	Colon	10.0	Well.	T3	No	No	No
21	62	F	Colon	6.5	Moderate	T3	No	No	Yes
22	91	F	Colon	5.5	Moderate	T3	No	No	No
23	72	F	Rectum	3.0	Moderate	T3	No	No	No
24	56	M	Colon	5.0	Moderate	T3	Yes	Yes	No
25	59	M	Colon	4.0	Moderate	T3	Yes	No	Yes
26	58	F	Rectum	2.5	Moderate	T2	No	No	No
27	75	M	Rectum	1.0	Moderate	T1	No	No	No
28	48	M	Colon	2.0	Moderate	T1	No	No	No

M: male, F: female.

**Figure 1:**
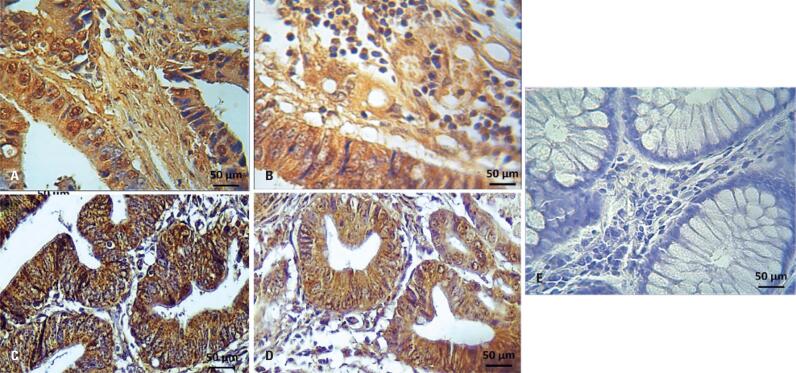
Protein expression of colorectal carcinoma biomarkers by immunohistochemistry (IHC). (A) Intensity of expression (ItE) of anti-CD44. (B) ItE of anti-CD133. (C) ItE of anti-ALDH1-2. (D) ItE of anti-LGR5. (E) Negative reaction control

In 28 colorectal cancer tissue samples, immunohistochemical reactions were performed for four antibodies to stem cells: anti-CD44, anti-CD133, anti-ALDH1, and anti-LGR5 (Figure 1). Table 2 presents the ItEs of CD44, CD133, ALDH1-2, and LGR5 proteins, as evaluated by immunohistochemical reactions with the primary antibodies.

**Table 2:** Relationship between the intensities of expression (ItE) of CD44, CD133, ALDH1-2, and LGR5 proteins in colorectal carcinoma tissue

Case	CD44*	CD133*	ALDH1-2*	LGR5*
1	100.80	146.68	207.61	187.16
2	177.46	152.42	177.4	213.3
3	102.56	81.66	186.1	203.2
4	177.06	150.74	159.4	213.1
5	165.72	105.64	196.1	193.5
6	175.70	152.56	233.00	218.21
7	171.36	188.82	174.20	220.25
8	186.54	154.70	220.58	216.68
9	161.34	88.36	212.28	172.53
10	170.44	138.80	185.45	160.33
11	164.94	86.28	212.73	200.75
12	134.72	126.58	140.08	179.90
13	153.26	63.36	167.60	203.45
14	149.62	100.84	207.85	207.53
15	202.82	139.50	157.60	184.93
16	189.52	102.34	209.23	180.23
17	168.32	179.24	180.22	213.54
18	148.28	95.94	156.73	170.80
19	146.18	60.62	146.15	187.27
20	168.44	72.56	204.03	185.10
21	158.16	53.54	182.73	153.38
22	150.36	80.36	200.48	168.93
23	169.48	104.78	203.50	192.85
24	195.26	169.20	191.15	173.55
25	132.98	161.68	153.03	150.10
26	125.80	86.52	184.28	154.88
27	50.74	49.72	51.20	50.70
28	170.50	95.28	167.30	188.68
Mean	156.01 (SD±31.94)	113.88 (SD±40.39)	181.00 (SD±334.87)	183.74 (SD±13.01)

* ItE in *μ*.o./*μ*m².

SD: standard deviation.

The images obtained by photomicrography were analyzed using the ImageJ Analysis System (Softium Informática, São Paulo, Brazil), adjusted to optical units per square micrometer (*μ*.o. /*μ*m²).

**Figure 2:**
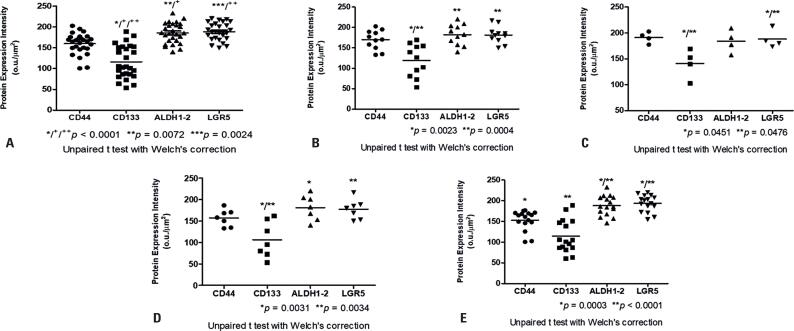
Distribution of protein expression intensity by staining with CD44, CD33, ALDH1-2, and LGR5 antibodies in colorectal (A), colon (B), left colon (C), right colon (D), and rectum (E) carcinoma tissues

When analyzing antibody expression in patients with colorectal cancer over 60 years of age, tissue levels of CD44, ALDH1-2, and LGR5 proteins were significantly higher than CD133 protein expression (p<0.05; Figure 2).

Immunohistochemistry (IHC) showed that ALDH1-2 and LGR5 protein levels were significantly higher in the neoplastic tissues of male (p<0.0001) and female (p<0.05) patients with CRC.

The expression of anti-CD44, ALDH1-2, and LGR5 antibodies demonstrated a significant increase in CRC tissues compared to anti-CD133 expression (p<0.05). In the colon, CD44 protein expression was significantly higher than that of CD133 protein expression, whereas ALDH1-2 and LGR5 protein expression was significantly higher than that of anti-CD133 (p=0.0004). The colon was separated into the right colon (cecum, ascending colon, and proximal two-thirds of the transverse colon) and left colon (distal third of the transverse colon, descending colon, and sigmoid colon). The staining of the proteins ALDH1-2 and LGR5 showed a significant increase in the right colon compared with the staining of CD44 (p=0.0031) and CD133 (p=0.0034). In the left colon, the staining intensity of CD44 was significantly higher than that of CD133 and LGR5 (p=0.0451), and that of LGR5 was significantly higher than that of ALDH1-2 (p=0.0476). In rectal carcinomas, the tissue levels of ALDH1-2 and LGR5, as determined by immunohistochemical staining, were significantly higher than those of CD44 (p=0.0003) and CD133 (p<0.0001) (Figure 2).

Concerning colorectal tumor size, LGR5 protein expression was significantly higher in all tumors measuring >6.0 cm in their longest axis than ALDH1-2, CD44, and CD133 protein expression (p<0.0001).

In tumors with moderate cellular differentiation, the expression of anti-CD44, ALDH1-2, and LGR5 antibodies was significantly higher than that of anti-CD133 (p<0.05).

In colorectal tumors with T2 intestinal parietal invasion, LGR5 protein expression was significantly higher than ALDH1-2, CD44, and CD133 protein expression (p<0.05). Moreover, in T3 intestinal parietal invasion, LGR5 and ALDH 1-2 protein expression was significantly higher than that of CD44 and CD133 (p<0.05).

**Figure 3:**
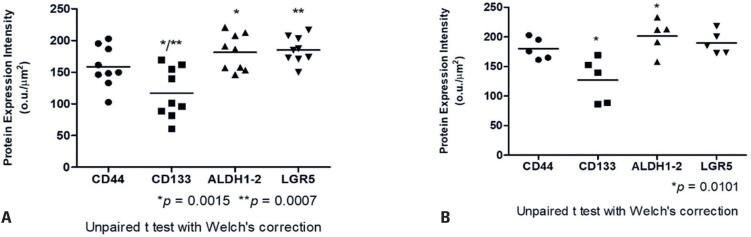
Distribution of staining intensity for CD44, CD33, ALDH1-2, and LGR5 in colorectal carcinoma tissue with tumors showing vascular (A) and perineural (B) invasion

ALDH1-2 and LGR5 protein expression significantly increased in the presence of vascular and perineural invasion relative to CD44 and CD133 protein expression (p<0.05; Figure 3).

**Figure 4:**
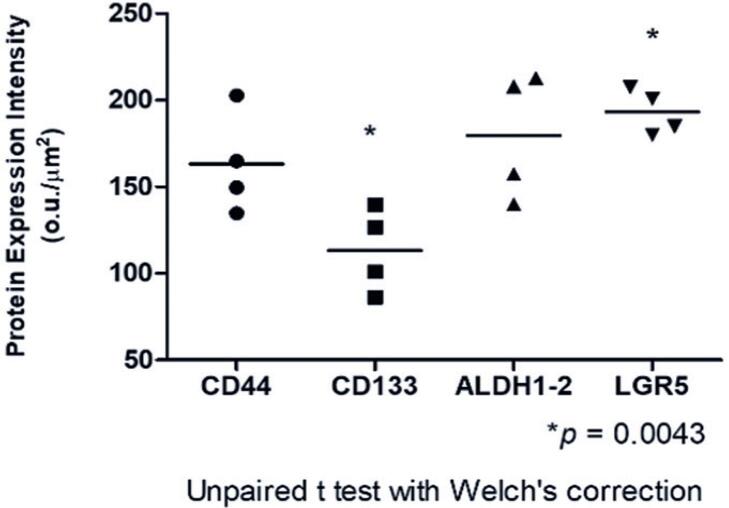
Distribution of the staining intensity for CD44, CD33, ALDH1-2, and LGR5 in colorectal carcinoma tissue with tumors showing neoplastic invasion of lymph nodes

LGR5 protein expression was significantly increased in lymph node metastasis compared to ALDH1-2, CD44, and CD133 protein expression (p<0.05; Figure 4).

## DISCUSSION

Increasing evidence suggests that a population of self-renewing tumor cells, known as CSCs, is responsible for tumor progression, recurrence, metastasis, and therapeutic resistance.^(34–36)^ Therefore, the identification of CSCs is crucial for identifying therapeutic targets and useful prognostic markers for CRC.^(22,37)^

The proteins CD44, CD133, ALDH1-5, and LGR5 have been considered potential stem cell biomarkers in various malignant tumors, including CRC.^(38,39)^

In the present study, CD44, ALDH1-2, and LGR5 proteins showed higher tissue levels in CRC than CD133 protein levels. This finding suggests that these markers, in general, have a greater potential for identifying stem cells in CRC tissue than the CD133 protein. ALDH was used to clarify the role of CD133 as a stem cell marker. For example, in hepatocellular carcinoma, the majority of ALDH+ cells are also CD133+, but there are many CD133+/ALDH− cells. Furthermore, CD133+/ALDH+ cells have the most significant tumor-forming potential, followed by CD133+/ALDH− and then CD133−/ALDH− populations.^(26,27)^

There was a significant predominance of immunohistochemical staining for ADLH1-2 and LGR5 in patients aged >60 years. Because most individuals with CRC are over 60 years of age, this finding suggests a stronger association between these markers and sporadic CRC cases, which represent most patients over 60 years of age. As a corollary of this result, we observed a dominance of tissue staining of ADLH1-1 and LGR5 proteins in both males and females, relative to CD44 and CD133 proteins.

CD44 and ALDH1-2 were significantly more abundant in the colon than LGR5 and CD133. However, when we separated the right from the left colon, we found that ALDH1-2 and LGR5 protein levels were significantly higher in the right colon. At the same time, the CD44 protein was also significantly predominant in the left colon. In rectal carcinoma tissues, ALDH1-5 and LGR5 proteins were stained at significantly higher levels than CD44 and CD133. These differences may be related to the embryological origin of the various segments of the large intestine and/or the distinct morphological and molecular characteristics of the cells within each segment. In all colorectal tumors measuring more than 1 cm in length, the study showed that tissue levels of ALDH1-5 and LGR5 were significantly higher than those of CD44 and CD133. This result reinforces the findings regarding the distribution of these proteins in CRC tissues and may indicate a greater characterization of potential stem cells.

All but one CRC cell line was moderately differentiated, and the tissue levels of CD44 and ALDH1-5 were higher than those of CD133. This finding suggests that these proteins, which are upregulated in carcinoma tissues, are promising for selecting potential stem cells in CRC.

The depth of tumor penetration into the intestinal wall was significantly associated with the proteins ALDH1-5 and LGR5, but not with CD44 and CD133. Therefore, it is possible to speculate that ALDH1-2 and LGR5 are associated with advanced CRC staging.

Tumors with or without vascular invasion showed significant staining for ALDH1-5 and LGR5 proteins relative to CD44 and CD133. This result may reflect higher levels of these proteins in CRC tissues.

In the presence or absence of perineural invasion, ALDH-1 was the only protein with significant staining relative to the other proteins, except for LGR5, in carcinoma tissues without perineural invasion. However, this protein, along with LGR5, showed increased levels in CRC tissues compared to CD44 and CD133. LGR5 protein levels were significantly elevated in all patients with lymph node metastases regardless of the location of the affected lymph nodes. In contrast, the ALDH1-5 protein was elevated only in the lymph nodes closest to and distant from the tumor.

High expression of the DNA repair mechanism of ALDH1 isoform 1 in CSCs may help overcome the effects of chemoradiotherapy, and ALDH1 could act as a strong prognostic marker in patients with CRC. In the present study, we observed that ALDH1-2 protein expression was significantly increased in patients with advanced CRC. This finding is significant because ALDH1-positive cells with CSC properties, such as differentiation, self-renewal, and tumorigenicity, are more resistant to chemoradiotherapy and are associated with poor cancer prognosis. Increased ALDH1 expression in colon cancer tissue samples was associated with poor differentiation (high grade) and metastasis. The detoxifying capacity of ALDH1 may contribute to the longevity of CSCs and protect them against oxidative damage.^(26,27,40)^

Furthermore, high ALDH1 expression correlates with the clinicopathological characteristics of CRC, such as T stage, N stage, and tumor differentiation^(41)^ but not with patient age, as observed in the present study. Moreover, ALDH1 was identified as an independent factor associated with decreased survival, which may be a consequence of the protection provided by this protein to stem cells. Moreover, ALDH1 acts as a promoter to induce epithelial-mesenchymal transition (EMT) in tumor cells. Epithelial-mesenchymal transition promotes epithelial neoplastic cells to attain stem cell status and is correlated with tumor invasion and metastasis.^(26,40)^

LGR5, a G-protein-coupled receptor with a leucine-rich repeat, is overexpressed in CRC cells, and its expression changes with CRC progression.^(42)^ LGR5+ cells are located at the base of the crypt and have the potential to generate all intestinal epithelial cell lineages, maintaining self-renewal and homeostasis of the intestinal mucosa.^(43)^ The mechanism of self-renewal regulation by LGR5+ occurs through the Wnt/β-catenin pathway.

CD133, known as the prominin-1 glycoprotein, organizes cell membrane topology. Initial studies to characterize CSCs in CRC began with the isolation of CD133+ cells. CD133+ cells produce IL-4 and use it to prevent apoptosis. When isolated from primary CRC samples, these cells were capable of forming tumors in mice, remained undifferentiated when cultured in serum-free media, and became more aggressive over generations. However, most tumors are composed of CD133− cells, which are incapable of initiating tumor formation. These findings are consistent with the results of the present study, which did not reveal a more pronounced level of CD133 than that of the other proteins evaluated in terms of the studied variables.^(15,19,44,45)^

CD44 has also emerged as an important marker of CRC stem cells. It is a cell surface glycoprotein that functions in cell–cell interactions, cytoskeletal adhesion to the extracellular matrix, and cell migration.^(46)^ CD44 transcription is at least partly activated by Wnt-β-catenin signaling, and its overexpression is an early event in the transformation of colorectal adenomas to carcinomas.^(47)^ In colonic mucosa, it is used as a marker of immature differentiation. A reduction in CD44 expression prevents tumorigenesis and clone formation.^(48)^ In the present study, CD44 protein was significantly linked to tissue expression and was the protein with the highest elevation in left colon carcinomas.^(13,19,22,23)^

Colorectal carcinoma is currently thought to originate from CSCs of the large intestine.^(49,50)^ Once the activities of the mutated genes in CRC have been identified, this knowledge may eventually facilitate the development of new approaches to cancer therapy, aiming to limit the proliferative capacity of these mutated clones, in addition to administering growth factor antagonists through gene therapy or immunotherapy targeted at specific cellular markers. The major challenge faced by researchers is to adapt treatments to minimize side effects on normal tissues, which can best be achieved by detailing the specific differences that exist between ordered and disordered cell growth.

The main limitation of this study is the small number of cases, which has the potential to bias the analyses. However, the statistical tests showed significance in the results presented. The lack of specific stem cell function assays may compromise the accuracy in identifying CSCs.

## CONCLUSION

Among the colorectal cancer stem cells marker proteins, tissue levels of ALDH1-2 and LGR5 were associated with greater tumor aggressiveness. These results also contribute to the selection of neoplastic stem cells in colorectal carcinoma. If the differences between cancer stem cells and other cells could be identified, a more effective targeted therapy for colorectal carcinoma could be implemented.

## Data Availability

The underlying content is contained within the manuscript.
